# A Neonatal Case of Communicating Cervical Esophageal Duplication Cyst

**DOI:** 10.7759/cureus.90877

**Published:** 2025-08-24

**Authors:** Nadia Boujida, Lina Belkouchi, Soukayna Jabour, Khadija Elaitari, Siham Elhaddad, Nazik Allali, Latifa Chat

**Affiliations:** 1 Radiology, Children's University Hospital, Rabat, MAR; 2 Department of Radiology, Children's Hospital of Rabat, Ibn Sina University Hospital Center, Mohammed V University, Rabat, MAR

**Keywords:** cervical cyst, congenital anomalies, esophageal duplication cyst, imaging diagnosis, neonate

## Abstract

Esophageal duplication cysts are rare congenital anomalies of the gastrointestinal tract, most frequently located in the thoracic esophagus, with cervical involvement being exceedingly uncommon. Diagnosis and management of these lesions, especially when presenting with atypical imaging features such as an air-fluid level, can be challenging. We report the case of a 21-day-old male neonate, admitted for evaluation of a left cervical swelling. Ultrasound and CT imaging revealed a left basi-cervical cystic lesion with internal air-fluid level, intimately related to the esophagus. A digestive contrast study demonstrated opacification of the cyst during contrast passage along the esophagus, confirming a communicating esophageal duplication cyst. The patient remained asymptomatic despite the lesion’s size and location.

## Introduction

Duplication cysts encompass a wide range of mass lesions that can occur throughout the gastrointestinal tract. Esophageal duplication cysts are rare congenital cystic lesions arising from errors in foregut budding during embryonic development, with a reported incidence of one in 8,200 autopsies [1]. Most esophageal duplication cysts are located in the thoracic region; cervical involvement is exceedingly rare. We report a rare case of a cervical esophageal duplication cyst in a 21-day-old male neonate.

## Case presentation

A 21-day-old male neonate, born at term, with a history of neonatal intensive care unit (NICU) admission for respiratory distress due to meconium aspiration, was brought in for evaluation of a left submandibular swelling. The parents reported that the mass had been intermittently noticeable since the first days of life. On examination, the neonate was found to be afebrile with failure to thrive. A soft, non-tender left lateral cervical mass was palpated, without signs of local inflammation. No palpable lymphadenopathy was noted.

**Figure 1:**
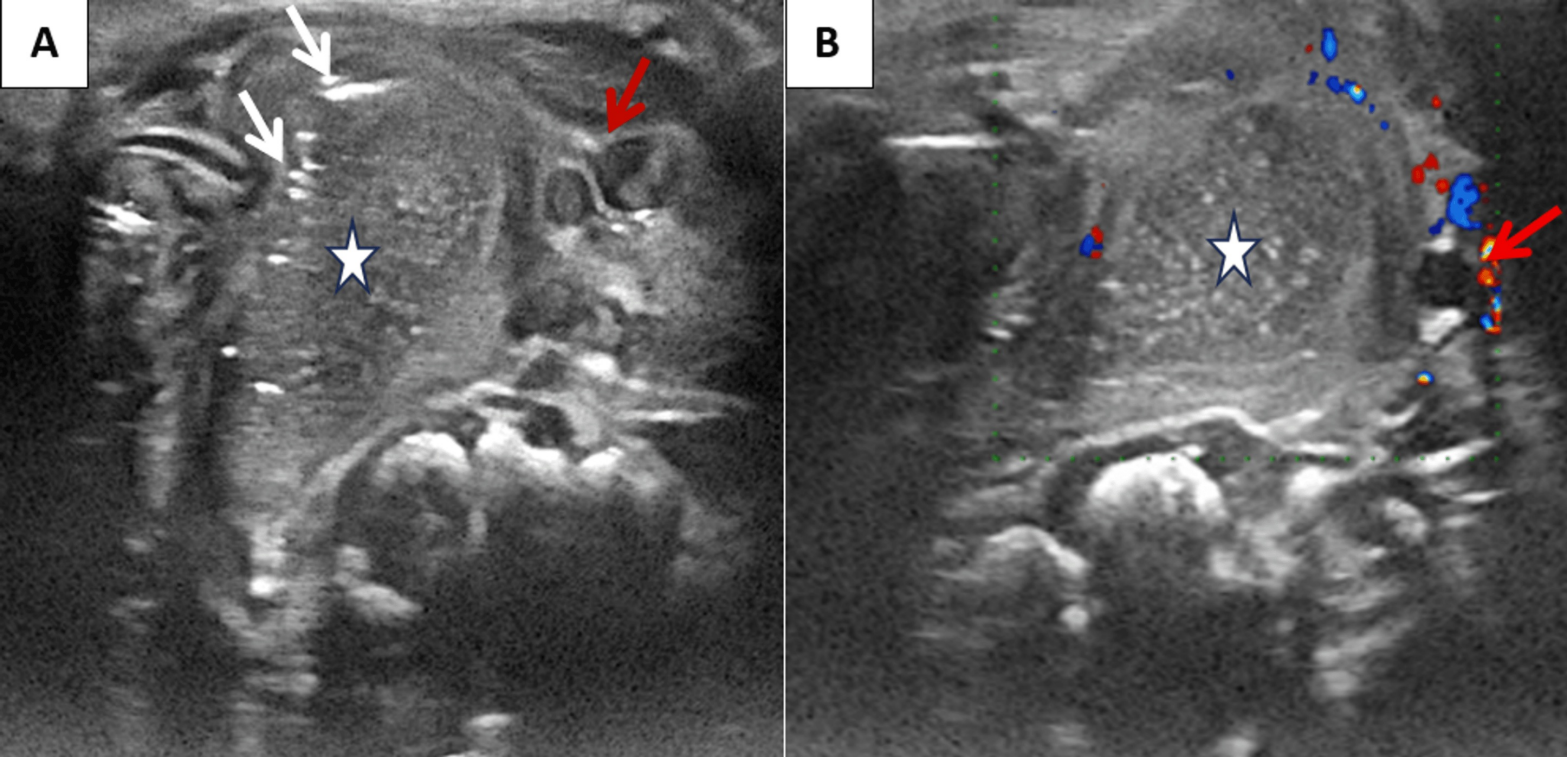
Ultrasound images in axial sections The images before (A) and after (B) color Doppler show a right lateral cervical mass (star) with fluid content generating a posterior acoustic shadow and containing hyperechoic foci consistent with air content (white arrow), showing no color Doppler uptake, and displacing the jugulocarotid axis laterally (red arrow).

Ultrasound examination demonstrated a left lateral cervical collection with a thickened wall and finely echogenic fluid content, measuring 24 × 15 mm. The collection contained hyperechoic foci consistent with air content. There was no color Doppler uptake. It was in close contact with the left thyroid lobe and the esophagus and displaced the ipsilateral common carotid artery and the internal jugular vein, which remained patent (Figure 1).

**Figure 2:**
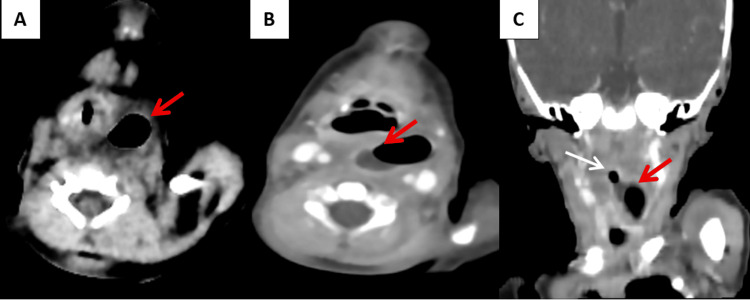
Cervical CT scan images Cervical CT scan without (A) and with contrast administration (B–C), in axial sections (A–B) and coronal reconstruction (C) reveals a well-defined left basi-cervical collection with an air-fluid level (red arrow). The collection exhibits a thickened wall with post-contrast enhancement and is in intimate contact with the esophagus (white arrow).

A cervical CT scan revealed a well-defined, left basi-cervical collection with hypodense fluid content and internal air bubbles. The collection measured 23 × 13 × 24 mm (transverse × anteroposterior × height). It exhibited a thickened wall with post-contrast enhancement and was in intimate contact with the esophagus and the left thyroid lobe. The collection remained separate from the tracheobronchial tree, and there was no evident fistulous tract communicating with the piriform sinus. The cervical vascular structures were preserved, as were the parotid and submandibular glands. No infiltration of the prevertebral muscles was observed (Figure 2).

**Figure 3:**
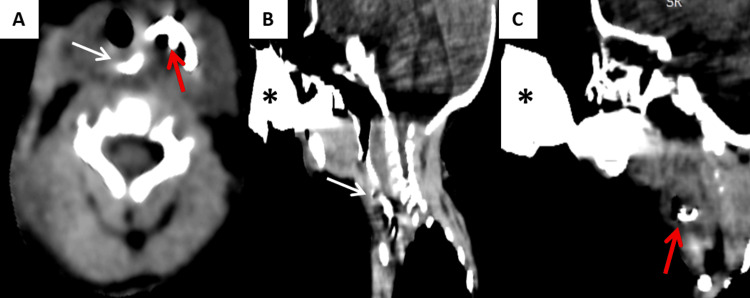
Cervical CT scan with oral contrast administered Cervical CT scan with oral contrast administered via feeding bottle (*), axial (A) and sagittal reconstructions (B–C) demonstrates opacification of the cervical collection (red arrow) during the passage of contrast along the esophagus (white arrow), confirming a communication between the esophageal lumen and the cervical cystic mass.

A digestive contrast study was performed using oral administration of Gastrografin prior to CT imaging. The examination demonstrated opacification of the cervical collection during the passage of contrast along the esophagus, confirming a communication between the esophageal lumen and the cervical cystic mass, findings compatible with a communicating digestive duplication cyst (Figure 3).

## Discussion

Although rare, duplications of the gastrointestinal tract most frequently involve the ileum, with the esophagus ranking as the second most common site. Approximately 23% of esophageal duplications affect the cervical segment. The cystic variant predominates, whereas tubular duplications are less frequent, comprising only 5-10% of cases. Notably, 60% of cystic duplications are situated in the distal third of the esophagus and are typically located along its right side [2].

Esophageal duplication cysts are believed to originate from errors in foregut budding during the third to sixth weeks of gestation. During embryologic development, the laryngotracheal groove divides into dorsal and ventral portions, which give rise to the esophagus and the respiratory tract, respectively. An esophageal cyst develops when secretory vacuoles fail to coalesce during the phase of foregut luminal obliteration. The timing of the budding error determines the cyst’s location: early developmental errors result in cysts forming within the mediastinum, such as cervical esophageal duplication cysts [3].

Esophageal duplications can present in three forms: a cystic type, which may or may not communicate with the esophageal lumen; a tubular form; or a diverticular form. Gastrointestinal tract duplications share three defining features: they maintain intimate contact with the adjacent intestinal tract; they are lined by mucosa similar to the corresponding segment of the digestive system; and they possess a smooth muscle layer, although cases of esophageal duplications lacking muscle fibers have been reported. In this case, the wall between the cyst and the esophagus was complete and included a smooth muscle layer. Approximately 80% of cysts do not communicate with the esophageal lumen, while the remaining cases typically run parallel to the esophagus and maintain communication with its lumen [2].

The majority of patients with esophageal duplication cysts are asymptomatic, with many lesions detected incidentally or identified prenatally through imaging studies. However, cysts located in the neck are more likely to produce clinical symptoms such as dysphagia, respiratory distress, cervical swelling, or even stridor, depending on the size and precise location of the lesion. In our case, the patient was found to have a cervical lesion prenatally but remained asymptomatic during the first six months of life [4].

Imaging examinations play a crucial role in the clinical diagnosis of esophageal duplication cysts. A chest radiograph may reveal space-occupying lesions and deviation of the trachea. On ultrasound, esophageal duplication cysts typically appear as an anechoic cystic lesion with posterior acoustic enhancement when the content is purely fluid. However, the cyst’s content may become finely echogenic to hyperechoic if it contains proteinaceous material or hemorrhage. A highly suggestive feature is the cyst wall’s layered appearance, showing an inner hyperechoic layer (mucosa), separated from an outer hyperechoic layer (serosa) by a thin hypoechoic band of smooth muscle, creating the so-called “muscular rim sign” or “double wall sign.” A Y-shaped configuration at the junction between the cyst and the digestive wall is considered more specific, though it requires a subserosal location, which is not always present. Esophageal duplication cysts can also be diagnosed prenatally by ultrasound as early as the fetal period [5].

On CT, duplication cysts typically appear as fluid-filled or fluid-air lesions, often with a thin wall that enhances after contrast administration and lies in close contact with a segment of the normal digestive tract. Oral contrast ingestion may help identify a communication between the cyst and the esophageal lumen when present. Occasionally, these cysts may demonstrate inflammatory wall thickening with a central fluid component, potentially mimicking a necrotic tumor. In hemorrhagic forms, the lesion may appear spontaneously hyperdense, raising suspicion for a solid mass [6].

Contrast esophagography remains a key examination for diagnosing communicating esophageal duplications [7]. In non-communicating cystic forms, it may reveal an extrinsic impression on the adjacent digestive segment and displacement of surrounding structures, though it rarely causes complete obstruction of contrast passage. In tubular duplications, a tubular structure communicating with the digestive lumen can be visualized [5].

On MRI, esophageal duplication cysts appear as cystic formations, typically featuring a thin wall that enhances after contrast administration and lying close to normal digestive structures. Oral contrast may help demonstrate communication when present. As with CT, inflammatory wall changes combined with a fluid center can mimic a necrotic tumor, while hemorrhagic cysts may appear spontaneously hyperintense, resembling solid tissue masses [5].

The presence of an air-fluid level within an esophageal duplication cyst, as seen in our case, is exceedingly rare. Cervical lesions exhibiting an air-fluid level are more commonly associated with abscesses, infected piriform fossa fistulas, or tracheal cysts. Consequently, cervical esophageal duplication cysts containing an air-fluid level are prone to misdiagnosis, which can lead to inappropriate therapeutic decisions. For example, while aspiration or drainage may be effective for treating an abscess, these interventions are unsuitable and potentially hazardous for an esophageal duplication cyst, as they can result in severe complications. This distinction is critical, given that esophageal duplication cysts carry risks of complications such as perforation, infection, hemorrhage, compression of surrounding structures, and even malignant transformation [4].

Regarding treatment, surgical resection remains the standard approach for esophageal duplication cysts. It is strongly recommended for all symptomatic lesions and is also advised for asymptomatic cysts to avert future complications [8]. Studies have consistently shown that recurrence is rare following complete surgical removal. Although malignant transformation within esophageal duplication cysts is uncommon, isolated cases have been reported in the literature [2]. Endoscopic resection can be a safe and effective option for cervical esophageal duplication cysts. When the lesion is easily visualized with a laryngoscope, this minimally invasive approach reduces the risk to deeper neck structures and avoids an external scar. Nonetheless, depending on the cyst’s exact location and the patient’s anatomy, an open surgical approach may still be necessary [9].

## Conclusions

This article presents the case of a 21-day-old male neonate, admitted for evaluation of a left cervical swelling. The patient was diagnosed with a communicating esophageal duplication cyst using ultrasound, CT imaging, and digestive contrast studies. Although rare, cervical esophageal duplication should be included in the differential diagnosis for children presenting with dyspnea, dysphagia, or neck masses. Complete surgical resection remains the definitive treatment.
